# Investigating Six Common Pesticides Residues and Dietary Risk Assessment of Romanian Wine Varieties

**DOI:** 10.3390/foods11152225

**Published:** 2022-07-26

**Authors:** Georgiana-Diana Dumitriu Gabur, Iulian Gabur, Elena Iulia Cucolea, Teodor Costache, Dan Rambu, Valeriu V. Cotea, Carmen Teodosiu

**Affiliations:** 1 Department of Environmental Engineering and Management, “Gheorghe Asachi” Technical University of Iasi, 700050 Iasi, Romania; diana.gabur@tuiasi.ro; 2 Faculty of Horticulture, Iasi University of Life Sciences, 700490 Iasi, Romania; vcotea@uaiasi.ro; 3 Department of Plant Science, Iasi University of Life Sciences, 700490 Iasi, Romania; 4 Scient Research Centre for Instrumental Analysis, Tancabești, 077167 Ilfov, Romania; iulia.cucolea@scient.ro (E.I.C.); teodor.costache@scient.ro (T.C.); dan.rambu@scient.ro (D.R.)

**Keywords:** wines, pesticide residues, chronic (long-term) dietary risk, QuEChERS, LC-MS/MS

## Abstract

The food and environmental safety debate extends to the use of pesticides in agriculture including the wine sector, which is one of the most intensive pesticide users across the agricultural sector. Pesticide utilisation is a common agricultural practice to protect fruits and plants from pathogens and insects while maintaining high production levels. Grapevine is generally a crop that is subject to intensive phytosanitary treatments, and therefore, it can be assumed that pesticide residues will accumulate in the vine-shoots and, later on, end up in the grapes and wines. The aim of this study was to determine the pesticide content in red, rosé, and white wines after phytosanitary treatments applied in the vineyard and their impact on long-term dietary risks. The following six pesticides were analysed: oxathiapiprolin, myclobutanil, iprovalicarb, tebuconazole, chlorantraniliprole, and acetamiprid. Samples were extracted using the QuEChERS (quick, easy, cheap, effective, rugged, and safe) method and analysed for the residues of pesticides by liquid chromatography-tandem mass spectrometry. Results indicated that the observed pesticides in the wine samples ranged between 0.05 and 0.75 ng/g. Dietary risks due to pesticide residues for women and men were evaluated using the estimated daily intake (EDI), hazard quotient (HQ), and hazard index (HI) of wines. The HQs and HIs did not surpass the 1 value (HQ, HI < 1) for both women and men, denoting that the concentrations of pesticide residues in these wine samples do not pose any immediate risk to consumers. Moreover, a pesticide residue intake model (PRIMo) model analysis was conducted, and the results suggest that European adult consumers have a low pesticide residue intake due to moderate wine consumption. However, pesticide residue intakes have been associated with several human health problems and high toxicity levels, therefore reliable analytical methods to monitor their presence in horticultural crops is crucial for clean and safe food products and healthy consumers.

## 1. Introduction

Wine is an extremely popular beverage around the world with a high financial significance and increasing consumption levels every year. The latest report of the International Organisation of Vine and Wine (OIV) [1] states that the global area of viticulture comprises 7.3 million hectares. Consumers in the European Union utilised 114 million hectolitres (mhL) of wine in 2021 (+3% in 2020), representing 48% of the world consumption. Romania is considered as a major wine producer and wine consumer worldwide, as in 2018, it obtained 4.5 million hectolitres of wine and consumed approximately 4.0 million hectolitres, respectively [1].

Grapes are an important source of polyphenols and other health beneficial compounds [2], however, grapevine is a very susceptible crop to a large number of pathogens. The fruits and aerial parts of the grapevine can become infected by diseases such as downy mildew (*Plasmopara viticola*), Botrytis rot (*Botrytis cinerea*), black rot (*Guignardia bidwellii*), Eutypa dieback (*Eutypa lata*), Phomopsis cane/leaf spot (*Phomopsis viticola*), powdery mildew (*Unicola necator*) or sour rot (*Aspergillus niger*, *Alternaria tenius*, *Botrytis cinerea*, *Cladosporium herbarum*, *Rhizopus arrihizus,* and *Penicillium spp*.) [3].

From an economical perspective, fungal diseases cause significant yield and grape quality losses in grapevine or can increase the production cost through the use of antifungal treatments. Major wine-producing areas use specific grape varieties and have independent climate/soil conditions and management practices. These makes pest and disease control a major problem for most grapevine growers that requires immediate specific intervention to keep the quality and quantity of the grapes constant. For example, French farmers use up to 13.5 treatments per season for their local varieties under a moderate downy mildew attack [4]. Downy mildew and powdery mildew can cause up to a total yield loss during a year of high disease pressure [3]. These diseases also affect the rate of photosynthesis and the maturity of grapes [5] and can cause offensive odours and sensual defects in wine [6]. Improper selection and poor management during technology changes can pose significant risks [7].

Various diseases caused by multiple pests as insects, or fungi and other agents challenge high quality grape production, and disease control has become an important vineyard management issue. The handling of pesticides in the management of vineyards and pest control can result in wine contamination with a high amount of pesticide residues [8]. Pesticides can also affect the physiological processes of grapes including limitations in photosynthesis and plant growth [9]. Therefore, the increased use of chemical pest-control methods in viticulture has become a major concern for human health and safety, as pesticide residues from grapes can end up in wine, and later in the human body.

In addition, vine growers are directly exposed to chemical compounds during the preparation and application of pesticides [10]. The drifting of pesticides near vineyards is often the subject of neighbourhood conflicts, as the potential impact of pesticides on human health is currently a major concern in most world-renowned wine-growing areas [11,12].

During vine production and harvest, small quantities of toxic pesticide compounds, called residues, can remain on or in the grapes. It is important to develop reliable analytical methods to monitor the presence of pesticide residues in horticultural crops and the final products for consumers [13].

In 2020, Eurostat published the report “Pesticide sales statistics”, in which there was a distribution of around 346,000 tonnes of pesticides in the Europe Union, where sixteen EU countries (Austria, Belgium, Czechia, Denmark, Germany, Ireland, France, Italy, Cyprus, Latvia, Hungary, the Netherlands, Portugal, Romania, Slovenia, and Sweden) contributed data regarding all major pesticide groups in 2011 and 2020. At an EU level, these countries recorded sales of 251,868 tonnes of active pesticide substances in 2011 and 233,509 tonnes in 2020. Portugal, Denmark, Romania, Belgium, and Ireland also declared sales that were at least 20% lower in 2020 than 2011.

According to the Green Deal, all European countries should aim at decreasing the impacts that pesticide residues could have on human health and the environment. Similarly, the EU Farm to Fork Strategy has set an ambitious target of reducing by 50% the use and potential health risks associated with chemical pesticide use in the European Union [14]. 

The FAO Joint /WHO Meeting on Pesticide Residues (JMPR), made up of an independent, international expert scientific group, carries out the risk assessment of pesticide residues in food. The JMPR sets Health Based Guidance Values to protect people from both acute (acute reference dose—ARfD) and chronic (acceptable daily intake—ADI) risks. The Pesticide Residue Intake Model (PRIMo) is a European validated risk analysis model developed by the European Food Safety Authority (EFSA) that evaluates the acute and chronic (long-term) health risks associated with chemical pesticide residue intake from processed foods, fruits, and vegetables for European populations [15].

To minimise the human health risks associated with pesticide residue intake through the consumption of food products, the European Union (EU) has set the maximum residue limits (MRLs) of pesticide residues tolerated in plant-derived products used for human consumption [16]. It has been reported that some technologies in the winemaking process can modify and decrease the pesticide residue amounts in wine [17]. Therefore, it is very important to develop a fast, selective, and sensitive analytical method that enables the measurement and quantification of trace amounts of pesticides in wine [18].

Sample preparation techniques utilised for pesticide analysis in fruits, vegetables, and drinks comprise head space solid-phase microextraction (HS-SPME), solid phase extraction (SPE), matrix solid-phase dispersion (MSPD), accelerated solvent extraction (ASE), microwave-assisted extraction (MAE), and QuEChERS (quick, easy, cheap, effective, rugged, and safe) with dispersive solid phase extraction. Anastassiades et al. [19] introduced a method for the determination of pesticides in fruit and vegetables with a high water content. The QuEChERS technique is the most common sample preparation method in pesticide analysis due to its simplicity, low-cost, high throughout, and less solvent requirement. This method has been validated for many pesticide residues in many foods and drinks and was recognised as an official method by the Official Analytical Chemist Association (AOAC) in 2007. Numerous QuEChERS modified protocols on pesticide residue analysis have been described in the literature as extraction solvents by using ethyl acetate or acetonitrile [20] and various sorbents or a mix of them in the clean-up procedure (PSA, GCB, C18) [21]. 

For this study, the major objective was focused on the detection of pesticide residues in red, rosé, and white wines of six common chemical compounds used in vineyards. Additionally, we assessed the dietary and health risks associated with pesticide residue intake for European adult populations by using the Pesticide Residue Intake Model (PRIMo). Pesticide residues from wine samples were extracted using the QuEChERS method and detected by liquid chromatography-tandem mass spectrometry. To the best of our knowledge, this is the first study on this topic, based on Romanian wines, of the chronic health risk assessment for pesticide residues in the framework of the maximum residue levels linked with the moderate wine consumption of European populations.

## 2. Materials and Methods

### 2.1. Reagents and Materials

The standards of pesticides were purchased from Dr. Ehrenstorfer GmbH (Augsburg, Germany). For acetamiprid, chlorantraniliprole, and iprovalicarb, the mix DRE-Q60 004725 of 10 µg/mL each was used, while for the other three pesticides, custom individual 10 µg/mL standard solutions in acetonitrile were used, also from Dr. Ehrenstorfer. Dilutions were conducted in acetonitrile to the desired concentrations, and then a final dilution was made by transferring 0.25 mL of the standard solution, adding 10 µL of the internal standard (TPP 5 µg/mL), and then 0.74 mL of ultrapure water. The Quick QuEChERS-Medium Cartridge was purchased from United Chemical Technologies (Bristol, PA, USA); the extraction salts including magnesium sulphate (MgSO_4_), sodium chloride (NaCl), acetonitrile, 2-propanol, acetone were from Merck KGaA (Darmstadt, Germany); formic acid (HCOOH; purity ≥ 95%) was from Fisher Chemical; and ammonium formate (HCO_2_NH_4_) from Sigma Aldrich (St. Louis, MO, USA). Methanol was supplied by HiPerSolv, VWR Chemicals (Radnor, PA, USA). A Kinetex biphenyl chromatography column (2.6 μm, 4.6 × 150 mm) was purchased from Phenomenex (Torrance, CA, USA) and ultrapure water was obtained by a Milli-Q water purification system from Millipore (Bedford, MA, USA).

### 2.2. QuEChERS Extraction

For the QuEChERS procedure, 10 mL of the sample was transferred to a polypropylene centrifuge tube (50 mL). On top of each sample, 10 mL of acetonitrile was added. The samples were vortexed for 30 s, and then the salt mix (4 g MgSO_4_ + 2 g NaCl) was added and shaken with a rotary stirrer for 1 min. The samples were centrifuged for 5 min at 5000 rpm. From the supernatant, 1 mL was taken with a syringe and filtered through the “Quick QuEChERS cartridge”. The small sample used for this clean-up step was a requirement of the Quick QuEChERS cartridge guidelines. Afterwards, 0.25 mL of the filtrate was added to a 2 mL vial over which 10 µL of the internal standard (TPP 5 µg/mL) was pipetted and 0.74 mL of ultrapure water was added. This final dilution step was implemented as liquid chromatography good practice to prevent chromatographic band dispersion due to solvent mismatch. Finally, the obtained extract was placed into an autosampler vial and injected into the LC-MS/MS system.

### 2.3. Equipment and Instrumental Parameters

The UHPLC Flexar chromatographic system (Perkin Elmer, Waltham, MA, USA) with a Kinetex biphenyl column (4.6 mm × 150 mm, 2.6 μm particle size) was used for the determination of pesticides. The detection was realised by an AB SCIEX Triple Quad 5500 mass spectrometer (Framingham, MA, USA). The results obtained were analysed using the SCIEX Analyst 2.0 software.

The preparation of the working solutions were as follows: (a) mobile phase A (MPA)—315.28 mg ammonium formate was transferred to a mobile phase bottle by washing with 1 L ultrapure water, then 1 mL formic acid was added; (b) mobile phase B (MPB)—315.28 mg of ammonium formate was transferred by washing with 0.02 L ultrapure water; then 0.98 L of methanol was added and homogenised. The HPLC pump was programmed with a flow rate of 1 mL/min and gradient elution as shown in Table 1.

The final injection volume was set to 20 μL. The sample compartment temperature was set at 10 °C and the column oven was kept at 33 °C. The ionisation source was maintained at 500 °C. The following gas parameters were used: curtain gas, 35; collision gas, 9; ion spray voltage, 3500 V; ion source gas 1, 60; ion source gas 2, 60. In order to acquire one transition for each analyte, HPLC analysis was conducted by positive electro-spray ionisation using retention time-scheduled multiple reaction monitoring (MRM). The obtained data are presented in Table 2. One transition was used for quantitation, while the second transition was used for the confirmation of the target analyte identity. The area ratio of the two transitions in the sample chromatogram was within 100 ± 20% of the average area ratio of the two transitions in the standard solutions chromatograms.

### 2.4. Samples

**Figure 1:**
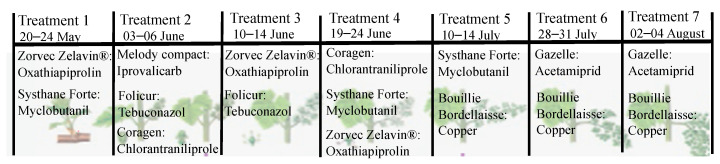
The phytosanitary treatments used at the vineyard (original figure).

Grapes were harvested, at maturity, in autumn 2020 from the 12 ha experimental vineyard located in the north-east part of Romania, at the Adamachi Farm, Iasi county, belonging to the Iasi University of Life Sciences “Ion Ionescu de la Brad” (IULS). The entire vineyard was exposed to seven treatments with phytosanitary compounds in the timeframe from May to August 2020. Pest control management in the vineyard was performed using the following pesticides: Zorvec Zelavin (oxathiapiprolin), Systhane (myclobutanil), Melody Compact (8.5% iprovalicarb + 40.6% copper oxychloride), Folicur (tebuconazole), Coragen (chlorantraniliprole), and Gazelle (acetamiprid). Treatments were carried out using the doses recommended by the manufacturers (Figure 1). 

The winemaking process was carried out at a laboratory scale at the Oenological Research Station of the Romania Science Academy, Iasi, Romania. Grape harvesting took place at the end of September when grapes were in good sanitary conditions. Grapes harvested from the vineyards were not washed and subjected directly to the winemaking process. The wines were obtained according to classical winemaking technologies. The vinified varieties were: Pinot Noir (red wine), Busuioaca de Bohotin (rosé wine), Feteasca Regala (white wine 1), Muscat Ottonel (white wine 2), Sauvignon Blanc (white wine 3), and Feteasca Alba (white wine 4). Sample wines were subjected to the conventional process, filtered, SO_2_ was added, bottled and stored in a dark cellar for further analysis. 

### 2.5. Risk Assessment

The long-term exposure was calculated to estimate the consumer health risks resulting from the residues in wines. The estimated daily intake of a pesticide residue was calculated as follows [22]:

(1) $$\mathrm{Estimated}\;\mathrm{daily}\;\mathrm{intake}=\frac{\mathrm{Concentration}\;\mathrm{of}\;\mathrm{pesticide}\;\mathrm{residue}\;\mathrm{in}\;\mathrm{wine}*\;\mathrm{consumption}\;\mathrm{of}\;\mathrm{wine}}{\mathrm{body}\;\mathrm{weight}}$$

The estimated daily intake of pesticide residues from wine consumption depends on the pesticide concentration, the consumption of wine, and the consumers’ body weight [23]. The estimated daily intake was calculated for the consumer groups of both women and men. For the dietary exposure assessment, the body weights of 72 kg and 150 mL/per person/per day for women and 85 kg and 250 mL/per person/per day for men have been recommended by the OIV [24] and Health Promotion and Disease Prevention Knowledge Gateway [25].

The hazard quotient (HQ) for all pesticides was determined from the estimated daily intake (EDI) to that residue divided by the acceptable daily intakes (ADI) [26]. The risk assessment of the cumulative exposure to pesticide residues was carried out by using the Hazard Index (HI) method. A value of HI >1 indicates that the non-carcinogenesis risk is not acceptable for the target population [27]. The calculations of HQ and HI are presented in Equations (2) and (3), respectively.

(2) $$\mathrm{Hazard}\;\mathrm{Quotient}\;\left(\mathrm{HQ}\right)=\frac{\mathrm{Estimated}\;\mathrm{daily}\;\mathrm{intake}\;\left(\mathrm{EDI}\right)}{\mathrm{Reference}\;\mathrm{value}\;\left(\mathrm{ADI}\right)}$$

(3) $$\mathrm{Hazard}\;\mathrm{index}\;\left(\mathrm{HI}\right)=\sum \mathrm{HQ}$$

#### Pesticide Residue Intake Model (PRIMo) Calculations 

MRL were obtained from the European Commission Regulations (see Table 5) and used as the residue input values based on the residues of pesticides detected in wines. All relevant information for the analysed pesticides were obtained from EU official sources. These data were used to estimate the dietary exposure to residual pesticides from wines.

The theoretical maximum daily intake (TMDI) for the studied wines was calculated using revision 3.1 of the European Food Safety Authority (EFSA) Pesticide Residue Intake Model (PRIMo) [15]. The PRIMo mathematical model for acute and chronic consumer exposure to pesticide residues found in wines is an internationally agreed risk assessment methodology. Wine pesticide residues detected with LC-MS/MS were introduced to PRIMo, with the specific parameters according to the EU MRL pesticide residue database. The data obtained estimated the exposure as the percentage of ADI and exposure (µg/kg bw per day) for adults of the EU population diets excluding diets for infants and children. The PRIMo model v 3.1 was used for the assessment of intake in relation to the consumption of processed food commodity, wine grapes/wine (code no 151020). 

Chronic (long-term) risk assessment for this study was based on JMPR methodology (IEDI/TMDI). The estimated chronic dietary exposure was compared to a specific compound concentration that can be consumed without a major negative human health impact.

### 2.6. Statistical Analyses

Cluster analyses was conducted to establish the differences between wines due to the pesticide residues quantified in wines. Statistical data processing and interpretation was carried out using Statgraphics Centurion XVI of StatPoint Technologies Inc. (Warrenton, VA, USA). The experimental data were collected in triplicate, and the experimental results are presented as the mean values and standard deviations.

## 3. Results

### 3.1. Method Validation

The monitoring of pesticide residues in white, rosé, and red wines was evaluated in the north-east of Romania by an optimised QuEChERS extraction method followed by UHPLC-MS/MS. The LC-MS/MS method accuracy was assessed using two quality control (QC) solution values. For the six pesticides analysed (Table 2), we observed a good average accuracy, ranging from 97.1 to 105.2%. These results show the good accuracy of the proposed methodology. The correlation coefficients (r) were higher than 0.99, which confirm the absence of curvature and the linearity of the analyte responses over the tested range of the wine standards. The standard concentrations corresponded to 2, 5, 10, 20, 30, and 50 ng/mL in sample units (peak area vs. concentration) for the pesticide residues. The chromatographic system injection was performed using the following steps: 1 × blank; 1 × standard solution μg/mL; 1 × QC1 solution; 1 × QC2 solution; 1 × sample solution; 1 × QC1 every 10 samples; 1 × QC1 after all samples; 1 × blank; 1 × wash. QC1 had the concentration of 5 ng/mL, while QC2 was 50 ng/mL.

The recovery of the spiked samples was performed for each of the analysed samples. The spike concentration was 5 ng/mL. The recovered concentration was calculated by subtracting the analyte concentration found in the sample from the analyte concentration found in the spiked sample. The recovery was then calculated by dividing the recovered concentration to the spiked analyte concentration. All of the spiked analyte recoveries were in the range of 70–130%. These results show the absence of a matrix effect for the target analytes.

The analyte detection was performed on a MS/MS instrument and multiple reaction monitoring (MRM) was employed. The specific transition for each analyte is available in Table 2 in Q1 (*m*/*z*) (parent ion) and Q3 (*m*/*z*) (fragment ion). The second transition monitored for each analyte was included in the revision. Additionally, in the section above Table 2, the use of the second transition for the verification of analyte identity was described. This is in accordance with the SANTE/12682/2019 guide requirements for MS/MS detection [28].

The LC-MS/MS assay was linear over the range 2–50 ng/mL (r2 > 0.99) for all of the studied pesticides. The samples used in the construction of the calibration curves presented an accuracy and precision comprised between 97.4 and 105.2% with a coefficient of variation (CV) in the range of 2–10.2%, respectively. The lower limit of quantification for each pesticide was determined as 2 ng/mL with an accuracy of 99.5–105.2%, as presented in Table 2. However, the lowest limit of quantification was not identified, as in this study, we did not investigate the concentration below the 2 ng/mL standard curve calibrator. The upper limit of quantification was set at 50 ng/mL and showed an accuracy of 99.9–101.4% and precision CV <2% (Table 2).

### 3.2. Screening of Pesticide Residues in Wine Samples

The method described in this study was implemented for the investigation of pesticide residues in six wine varieties (one red wine-Pinot Noir, one rosé wine-Busuioaca de Bohotin and four white wines—Feteasca Regala, Muscat Ottonel, Sauvignon Blanc, and Feteasca Alba). Considering the pesticide residues detected in wines, their analysis is required to ensure safe human consumption and limited exposure to health risks. 

The damage to human health and ecosystems caused by the presence of pesticide residues in agricultural products is one of the world’s most important food safety concerns. Efforts have been made primarily to use low toxicity fungicides, but it seems necessary to monitor this in the vineyard and market circulation of wines. Residual levels of pesticides and MRLs in agricultural products can have a significant impact on trade [29]. Therefore, it is necessary to monitor the generation and exact amount of pesticide residues in foods to protect consumers from exposure to dangerous chemicals. 

In the present investigation, six common pesticide residues were determined in wine samples produced by the Oenology laboratory from IULS in Romania, and the results were subsequently compared to the MRL values. The QuEChERS-LC-MS/MS method developed in the current study were independent of the pesticide concentration values and could simultaneously detect and measure more than 10 or more pesticide residues.

**Figure 2:**
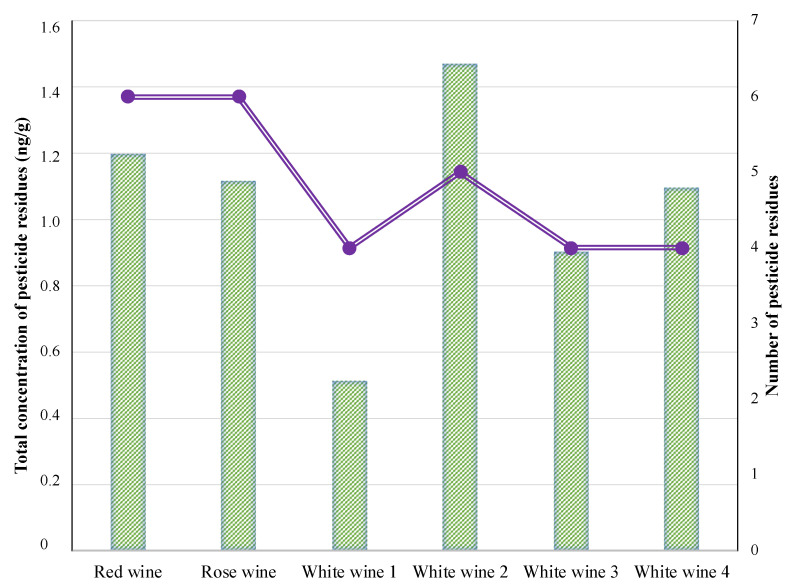
The total concentration and number of pesticide residues in each wine sample (original).

Figure 2 presents an overview of the total concentration of pesticide residues in all six wines and the number of pesticide residues found in each wine. All of the tested samples contained quantifiable residues of more than four pesticides. Both red and rosé wine samples contained traces of pesticide residues from all of the analysed pesticides. Interestingly, the sample from white wine 1 had four of the six pesticides and showed the lowest overall concentration, which was under 0.6 ng/g. The remaining samples showed traces of five or less pesticide residues: five pesticides in white wine 2 (Muscat Ottonel) and four pesticides in white wine 3 (Sauvignon Blanc) and white wine 4 (Feteasca Alba). However, no causal relationship could be established between the number of pesticides and the type of wine samples.

### 3.3. Pesticide Residues in the Wines

The pesticides present in the sample were extracted in acetonitrile and purified with the solid-phase extraction cartridge using the QuEChERS method. Then, the resulting sample was injected into the HPLC system where the compounds present were separated by high performance liquid chromatography following passage through a chromatographic column filled with the biphenyl reverse stationary phase. Following the elution step, the samples were transferred to the mass spectrometer where they were analysed based on the specific mass transition of each pesticide. Table 3 presents the results obtained for the analysis of the six pesticide residues in the white, rosé and red wine samples. 

Acetamiprid, N-[(6-chloropyridin-3-yl)methyl]-N′-cyano-N-methylethanimidamide, is part of a new class of insecticides that were designed in the 1980s, the neonicotinoids. The detailed structure of acetamiprid is that of a chloronicotinyl compound and it has been demonstrated to have strong negative stimulation of the nicotinic acetylcholine receptors in insects. The main use of acetamiprid is to control insects (aphids) that attack and impair leafy plants. The concentration of acetamiprid ranged between 0.05 ng/g in white wine 4 and 0.09 ng/g in white wine 1, therefore below the MRL (500 ng/g). Schusterova et al. [30] found that a concentration of acetamiprid ranging from 76 to 138 ng/g in grapes was detectable, while there was none in the wines.

Chlorantraniliprole, 3-bromo-N-[4- chloro-1-(3-chloro-2-methyl-6-(methylcarbamoyl)phenyl]-1-(3-chloro- pyridin-2-yl)-1,4-pyrazole-5-carboxanilide, is a novel insecticide belonging to the anthranilic diamide class of chemistry. This compound has a new mode of action for synthetic insecticides, named ryanodine receptor activators [31]. Chlorantraniliprole is being developed by DuPont Crop Protection and Syngenta Crop Protection and is considered for the control of Coleopteran, Lepidopteran, and Dipteran pests in grape.

The residues of chlorantraniliprole were largely below the MRL (1000 ng/g) in all of the trials. The highest value was 0.55 ng/g (red wine). In 2012, Malhat [32] reported that a study on the residue amount decreased to 1.7 ng/g in grape within the first 24 h after application. After this time interval, is seems that the chlorantraniliprole residues in grapes decreased to 1.09, 0.49, 0.18, and 0.05 ng/g at 3, 7, 10, and 15 days after spraying, respectively. The residues of this pesticide were at very low levels in grape, making them undetectable after 21 days post-plant treatment. In addition, Caboni et al. [33] described that sun exposure influenced the chlorantraniliprole residues to reminisce and increased the possibility of persisting residues in the final products. Until now, other studies on de chlorantraniliprole residues in wines have not been found.

Iprovalicarb, propan-2-yl N-[(2S)-3-methyl-1-[1-(4-methylphenyl)ethylamino]-1-oxobutan-2-yl]carbamate, is a fungicide that is usually used in vineyards to fight numerous fungal diseases such as *Plasmopara viticola*, and acts on the cellulose synthase complex in cell walls. The residual concentration of iprovalicarb in our study was between 0.07 and 0.75 ng/g, therefore below the MRL (2000 ng/g). In contrast, higher residual concentrations of 2–59 ng/g iprovalicarb were found by Schusterova et al. [30]. Additionally, Angioni et al. [34] found residue concentrations of 360 ng/g in red wines and 570 ng/g in white wines. Gonzalez-Rodrıguez et al. [35] reported that iprovalicarb residues could be absorbed by the grapevine must and decreased to almost 50% compared to the wine.

Myclobutanil is a chiral triazole fungicide with the chemical name 2-(4-chlorophenyl)-2-(1,2,4-triazol-1-ylmethyl)hexanenitrile. Normally, it is used to control fungal diseases that may occur in grapes and fruits [36]. It acts against fungi classified as *Ascomycetes*, *Deuteromycetes*, and *Basidiomycetes*, which provoke numerous diseases during grape growth. It decomposes under light exposure and aqueous solutions; however, it shows high hydrolysis stability at pH 5–9. Myclobutanil does not decompose under anaerobic conditions. The residues of myclobutanil were largely below the MRL (100 ng/g) in all of the samples. The values in wines ranged between 0.13 ng/g in white wine 2 and 0.15 ng/g in rosé wine. Moreover, some authors [37] have reported the presence of metalaxyl, myclobutanil, tebuconazole, and benalaxyl in wines of Spanish origin. Carpinteiro et al. [37] found 0.35 ng/g of myclobutanil in red wine and Pelajic et al. [38] in one sample of wine discovered 0.44 ng/g. 

Tebuconazole, 1-(4-chlorophenyl)-4,4-dimethyl-3-(1H-1,2,4- triazol-1-ylmethyl)pentan-3-ol), is a triazole fungicide used against leaf spot diseases. This compound is involved in the inhibition of ergosterin biosynthesis in fungi and acts as an efficient soil-borne and foliar fungal pathogen control measure [39]. It was listed among the top 10 active ingredients in the EU–fungicides report is used on several crops, especially in grapes. The concentration of tebuconazole was lower than 0.18 ng/g in wines, therefore below the MRL (1000 ng/g). This pesticide was identified in three samples of wines. 

Oxathiapiprolin, (1-(4-(4-((5RS)-5-(2,6-difluorophenyl)-4,5- dihydro-1,2-oxazol-3-yl)-1,3-thiazol-2-yl)-1-piperidyl)-2-(5-methyl-3-(trifluoromethyl)-1H-pyrazol-1-yl)ethanone), was the first of the piperidinyl thiazole isoxazoline fungicides to be discovered and was developed by the DuPont Company in 2007 [40,41]. This was developed as an phytosanitary treatment for oomycete and acts efficiently against many oomycetes including *Phytophthora infestans*, *Phytophthora capsici*, *Phytophthora sojae*, *Phytophthora parasitica,* and *Phytophthora nicotianae*. 

Oxathiapiprolin led to a final amount of pesticide residues that was much lower than the permitted MRLs (700 ng/g) in the samples. The concentrations were 0.15 ng/g in red wine and 0.14 ng/g un rosé wine. This pesticide was identified and quantified only in two wines, red and rosé wines, and was not detected in the white wine samples. Pingzhong et al. [42] found that the maximum final residue in grapes at the two experimental sites was less than 10 ng/g. This result suggests that grape harvesting could be performed in safe condition after 28 days if the recommended doses of oxathiapiprolin are used. Another author, Xiaohu et al. [43] found that the oxathiapiprolin could be detected but at concentration below the LOQs.

### 3.4. The Treatment Frequency Index (TFI)

**Figure 3:**
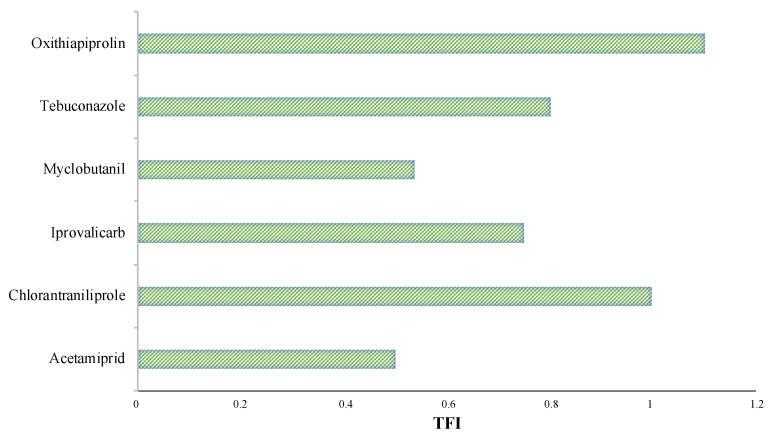
The distribution of the Treatment Frequency Index (TFI) for the studied arable cropping systems.

The Treatment Frequency Index (TFI) represents a measure for quantifying the total amount of pesticide applied for pest-control management across multi-year, multi-location sites. TFI is frequently used by vine growers as a tool to correlate the quantity of pesticides needed for the efficient treatment of high pest levels. THI offers significant details of the potential human and environmental risks linked to standardised pesticide use in farms. Moreover, TFI considers the pesticides’ active substance impact on a phytosanitary treated agricultural area. However, a major downside of TFI is that it does not estimate the toxic potential of pesticides, which can have a negative effect on human health or the environment. Different phytosanitary treatments may generate similar TFI due to the fact that pesticide active substances have different and various homologated doses. Therefore, no direct link could be identified between the number of pesticide doses applied at a farm and the TFI values [44]. In the investigated study, the TFIs were below a mean of 1 with the exception of oxathiapiprolin, which presented a value of 1.1. (Figure 3).

### 3.5. Statistical Analysis

**Figure 4:**
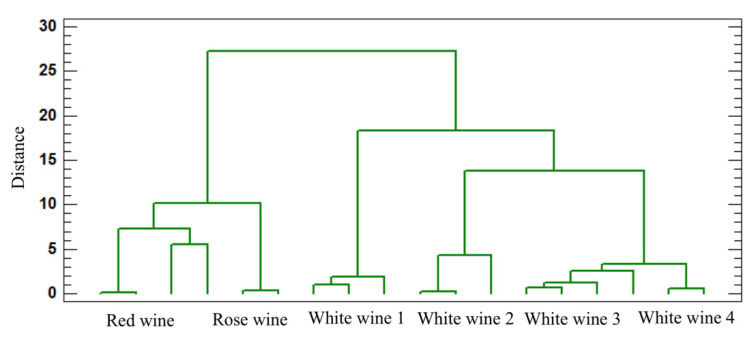
The cluster analysis according to Ward’s method of pesticide residues.

In order to better visualise the relative distribution of the pesticide residues, a cluster analysis was performed (Figure 4) that separated the wine samples into clusters in terms of their nearness or similarity. The squared Euclidean distance distinguished between the series’ similarity measurement. A smaller distance indicates the highest degree of relationship; therefore, those samples were grouped to the same aromatic series. Two clusters were formed, the first one included red and rosé wines and in the second cluster were all the white wines samples, to differentiate the wines according to their types. Therefore, the data obtained suggest that the maceration stage, the winemaking stages of each type, and the style of wine are the factors with a high influence on the pesticide residues.

### 3.6. Risk Assessment

The health risk characterisation was calculated based on the mean results. The EDI and HQ values in the female and male consumers are indicated in Table 4. The HQ values in the female and male consumers were lower than 1 due to the presence of all pesticide residues in the wines. The EDIs estimated the daily intake of pesticide residues from wine consumption in relation to the pesticide concentration for the consumer groups of 72 kg body weight and 150 mL/per person/per day for women and 85 kg body weight, and 250 mL/per person/per day for men, respectively. The results obtained were in the range from 1.05 × 10^−4^ to 9.33 × 10^−4^ mg/kg bw/day for women and 1.26 × 10^−3^ to 6.48 × 10^−4^ mg/kg bw/day for men. The HQs were in the range of 1.01 × 10^−2^ to 9.54 × 10^−2^ for women and 1.01 × 10^−1^ to 9.53 × 10^−3^ for men. 

For the red wines, the highest HQ for chlorantraniliprole and acetamiprid were 7.29 × 10^−4^ and 5.81 × 10^−3^ for women, respectively, and for acetamiprid, it was 8.20 × 10^−3^ for men. Higher HQs (7.87 × 10^−3^, 6.50 × 10^−3^, 5.98 × 10^−4^) were observed for tebuconazole, acetamiprid, and chlorantraniliprole in rosé wines for women, and for men, the HQs (9.18 × 10^−3^, 8.45 × 10^−4^) for acetamiprid and chlorantraniliprole. In the case of white wines, higher HQ values were observed for acetamiprid (7.81 × 10^−3^), tebuconazole (6.75 × 10^−3^), and myclobutanil (9.54 × 10^−2^) for women and tebuconazole (9.53 × 10^−3^), chlorantraniliprole (8.10 × 10^−4^), and acetamiprid (7.16 × 10^−3^) for men (Table 4). 

The dietary risk assessment of the six common pesticide residues identified in the six different wine varieties showed that the HQs for the pesticides did not surpass the unit value (HQ <1). Hence, the pesticide residues (Table 5) in all types of wines do not pose any health issues and are safe for human intake.

The HI values representing the cumulative risk assessment of the six common pesticide residues identified in various wine samples are shown in Table 4. The HI values for all of the quantified pesticides in women and men were calculated as 4.93 × 10^−2^ and 6.96 × 10^−2^ in Pinot Noir, 5.60 × 10^−2^ and 7.91 × 10^−2^ in Busuioaca de Bohotin, 2.92 × 10^−2^ and 4.12 × 10^−2^ in Feteasca Regala, 1.28 × 10^−1^ and 1.80 × 10^−1^ in Muscat Ottonel, 8.81 × 10^−2^ and 1.24 × 10^−1^ in Sauvignon Blanc, and 1.11 × 10^−1^ and 1.57 × 10^−1^ in Feteasca Alba.

The dietary risk assessment of the pesticide residues in all wines revealed a HI < 1, which indicated that the pesticide residues identified in the six wine varieties did not increase any of the health risks and the analysed wine should be considered as fit for human consumption. Our results are similar to a recently published article that investigated the acceptable safety risk of the pesticide residues from the wines and obtained HI < 1 [45].

In conclusion, there is no reason for wine consumers to be concerned about low- to medium-term exposure to residues through the consumption of the analysed wines, however, a long-term assessment should be conducted.

### 3.7. Chronic (Long-Term) Risk Assessment

This study investigated the dietary exposure to red, rosé, and white wines using the assumption of residue levels according to the European Union maximum residue limits (MRL) and the theoretical maximum daily intake (TMDI) approach [46]. Calculations were performed using the median residue levels from the supervised field trial data (STMR) obtained from the LC-MS/MS detection of pesticide residues in wines. 

The PRIMo tool (version 3.1) was used for the six pesticide residues in the red, rosé, and white wine samples in order to evaluate the long-term human chronic dietary exposure. The acceptable daily intake (ADI, in percent) and exposure (Exp, in µg/kg bw per day) for each pesticide are presented in Table 6. 

The results of long-term chronic exposure for the European population, considering the analysed pesticide residues in wines, indicate that, for most chemical compounds, the ADIs were below 1% and the exposure was lower than 1 µg/kg bw per day. However, iprovalicarb showed higher values for rosé wines, 3.2% for PT general, and an added high risk for white wines in general (e.g., PT general: white wine 2—ADI 12.5%, white wine 3—ADI 8.6%, and white wine 4—ADI 11.4%). Similar trends were observed for all of the other population groups. However, even though no residue exceeded 100% of the ADI for adults, one should consider the possibility of daily intake from other sources. The consumption of moderate wine quantities does not represent a potential chronic risk for the consumer groups investigated within the study. 

## 4. Conclusions

This study presents the results of the chromatography analyses of six common pesticide residues generally found in white, rosé, and red Romanian wines. Analytical methods detected six common pesticide residues (acetamiprid, chlorantraniliprole, iprovalicarb, myclobutanil, tebuconazole, oxathiapiprolin) used in vineyards. A modified QuEChERS method was used to extract the wine samples and the detection of pesticide residues was conducted by liquid chromatography-tandem mass spectrometry. The concentration levels of the detected pesticides in various wines ranged between 0.05 and 0.75 ng/g. However, none of the analysed samples exceeded the maximum residue levels (MRL), as recommended by the legislator in EU countries.

The dietary risk assessment for women and men showed that the HQs and HI for the pesticides detected did not surpass level 1. The chronic dietary intake of pesticide residues for the EU diets, using the mathematical approaches from PRIMo rev. 3.1, showed 1.87 mg/kg bw/day, or 12.5% of the ADI for PT adults. Indicative probabilistic calculations indicate that the actual chronic dietary exposure is likely to be even lower in general for most EU populations. This suggests that the pesticide residues in the analysed types of wines do not pose any direct consumer risk in the short-, medium-, and long-term. However, dietary risk assessment is of utmost significance to guarantee the rational and safe application of pesticides and minimise the potential health human risks. Therefore, the phytosanitary treatment application should be monitored constantly in order to ensure that consumers have access to safe and clean wine products.

## Figures and Tables

**Table 1:** The gradient elution program.

Time (min)	MPA %	MPB %
0.70	95	5
1.00	50	50
1.50	40	60
2.50	22	78
4.00	12	88
10.00	8	92
12.00	0	100
24.00	0	100
25.00	95	5
29.50	95	5

Note: MPA—mobile phase A, MPB—mobile phase B.

**Table 2:** The parameters for the analysis of the targeted pesticides in wine determined by LC-MS/MS.

Pesticides	Q1 (*m*/*z*)	Q3 (*m*/*z*)	Retention Time	R^2^	LOQ (ng/g)	LOD (ng/g)	Regression Equation	Mean Calculated Concentration (ng/mL)	Accuracy (%)	Standard Deviation (ng/mL)	Coefficient of Variation (CV %)	Response Factor
Acetamiprid	223.2	126.0	4.44	0.9942	0.40	0.12	y = 0.933 × + −0.192	2.46	99.5	0.08	3.2	2.93
								4.98	101.0	0.40	8.0	3.41
	Confirmation transition:							9.92	100.5	0.19	1.9	2.96
								19.35	98.2	1.13	5.9	3.37
	223.200	90.0						29.76	100.5	1.68	5.6	3.22
								49.41	100.2	1.39	2.8	3.46
Chlorantraniliprole	481.9	283.9	5.26	0.9948	0.22	0.07	y = 0.906 × + −0.253	2.54	100.1	0.06	2.2	0.87
								5.07	100.0	0.52	10.2	1.04
	Confirmation transition:							10.17	100.7	0.44	4.3	1.00
								19.95	98.3	0.93	4.7	1.01
	481.9	450.9						30.72	101.1	1.73	5.6	1.06
								50.66	99.9	1.82	3.6	1.08
Iprovalicarb	321.2	119.1	5.07	0.9944	1.62	0.04	y = 0.113 × + −0.0535	2.58	102.8	0.17	6.6	3.54
								5.04	100.2	0.44	8.6	4.12
	Confirmation transition:							10.00	99.1	0.33	3.3	3.52
								19.57	97.4	1.35	6.9	4.04
	321.2	91.1						29.72	98.7	1.48	5.0	3.95
								51.23	101.8	1.85	3.6	4.14
Myclobutanil	289.1	70.1	5.41	0.9900	0.74	0.22	y = 0.298 × + −0.125	2.52	100.1	0.19	7.6	0.46
								5.03	100.1	0.39	7.8	0.44
	Confirmation transition:							10.17	100.7	0.25	2.5	0.41
								19.94	99.2	0.85	4.2	0.47
	289.1	125.2						30.06	99.5	1.70	5.7	0.48
								50.53	100.5	1.62	3.2	0.48
Tebuconazole	308.2	70.0	5.58	0.9930	0.59	0.17	y = 0.132 × + −0.0859	2.62	105.2	0.20	7.6	0.35
								4.91	98.4	0.39	7.9	0.4
								9.68	97.1	0.36	3.7	0.35
								19.58	98.4	0.80	4.1	0.42
								29.76	99.5	1.79	6.0	0.39
								50.61	101.4	1.94	3.8	0.42
Oxiathiapiprolin	540.0	500.0	6.67	0.9924	1.58	0.47	y = 0.183 × + −0.144	2.63	103.9	0.16	6.1	0.24
								4.94	97.4	0.38	7.7	0.32
	Confirmation transition:							10.11	100.1	0.32	3.1	0.3
								19.73	97.2	1.31	6.7	0.34
	308.2	125.1						30.60	100.6	2.11	6.9	0.35
								51.00	100.8	2.75	5.4	0.36

In Table 2, the accuracy (%) is the average of the ratio of the calculated concentration over the theoretical standard solution concentration for five standard replicate injections for each calibration level.

**Table 3:** The concentrations of the pesticide residues (ng/g) found for the red, rosé, and white wine samples.

Pesticides	Red Wine	Rosé Wine	White Wine 1	White Wine 2	White Wine 3	White Wine 4
Acetamiprid	0.0697 ± 0.03 ^ab^	0.0781 ± 0.03 ^ab^	0.0937 ± 0.02 ^b^	0.0608 ± 0.02 ^ab^	0.0556 ± 0.01 ^a^	0.0504 ± 0.02 ^a^
Chlorantraniliprole	0.5456 ± 0.09 ^c^	0.4480 ± 0.08 ^b^	0.2156 ± 0.01 ^a^	0.4296 ± 0.04 ^b^	0.1943 ± 0.02 ^a^	0.2203 ± 0.02 ^a^
Iprovalicarb	0.1216 ± 0.07 ^a^	0.1901 ± 0.07 ^b^	0.0729 ± 0.00 ^a^	0.7509 ± 0.02 ^e^	0.5164 ± 0.01 ^c^	0.6871 ± 0.01 ^d^
Myclobutanil	0.1364 ± 0.01 ^ab^	0.1515 ± 0.00 ^c^	0.1314 ± 0.00 ^ab^	0.1309 ± 0.00 ^a^	0.1379 ± 0.01 ^b^	0.1379 ± 0.00 ^b^
Tebuconazole	0.1771 ± 0.01 ^c^	0.1134 ± 0.00 ^b^	nd	0.0972 ± 0.00 ^a^	nd	nd
Oxathiapiprolin	0.1466 ± 0.00 ^b^	0.1354 ± 0.00 ^a^	nd	nd	nd	nd

Note: Different letters (^a, b, c, d, e^) show significant differences at the 95% confidence level between the wine samples; nd—not detected.

**Table 4:** The estimated daily intake and hazard quotient in the red, rosé, and white wines.

Pesticides	Red Wine		Rosé Wine		White Wine 1		White Wine 2		White Wine 3		White Wine 4	
	Women	Men	Women	Men	Women	Men	Women	Men	Women	Men	Women	Men
	Estimated Daily Intake (EDI) (mg/kg bw/day)											
Acetamiprid	1.45 × 10^−4^	2.05 × 10^−4^	1.63 × 10^−4^	2.30 × 10^−4^	1.95 × 10^−4^	2.76 × 10^−4^	1.27 × 10^−4^	1.79 × 10^−4^	1.16 × 10^−4^	1.63 × 10^−4^	1.05 × 10^−4^	1.48 × 10^−4^
Chlorantraniliprole	1.14 × 10^−3^	1.60 × 10^−3^	9.33 × 10^−4^	1.32 × 10^−3^	4.49 × 10^−4^	6.34 × 10^−4^	8.95 × 10^−4^	1.26 × 10^−3^	4.05 × 10^−4^	5.71 × 10^−4^	4.59 × 10^−4^	6.48 × 10^−4^
Iprovalicarb	2.53 × 10^−4^	3.58 × 10^−4^	3.96 × 10^−4^	5.59 × 10^−4^	1.52 × 10^−4^	2.14 × 10^−4^	1.56 × 10^−3^	2.21 × 10^−3^	1.08 × 10^−3^	1.52 × 10^−3^	1.43 × 10^−3^	2.02 × 10^−3^
Myclobutanil	2.84 × 10^−4^	4.01 × 10^−4^	3.16 × 10^−4^	4.46 × 10^−4^	2.74 × 10^−4^	3.86 × 10^−4^	2.73 × 10^−4^	3.85 × 10^−4^	2.87 × 10^−4^	4.06 × 10^−4^	2.87 × 10^−4^	4.06 × 10^−4^
Tebuconazole	3.69 × 10^−4^	5.21 × 10^−4^	2.36 × 10^−4^	3.33 × 10^−4^	-	-	2.02 × 10^−4^	2.86 × 10^−4^	-	-	-	-
Oxathiapiprolin	3.05 × 10^−4^	4.31 × 10^−4^	2.82 × 10^−4^	3.98 × 10^−4^	-	-	-	-	-	-	-	-
	Red wine		Rosé wine		White wine 1		White wine 2		White wine 3		White wine 4	
	Women	Men	Women	Men	Women	Men	Women	Men	Women	Men	Women	Men
	Hazard Quotient (HQ)											
Acetamiprid	5.81 × 10^−3^	8.20 × 10^−3^	6.50 × 10^−3^	9.18 × 10^−3^	7.81 × 10^−3^	1.10 × 10^−2^	5.07 × 10^−3^	7.16 × 10^−3^	4.63 × 10^−3^	6.54 × 10^−3^	4.20 × 10^−3^	5.94 × 10^−3^
Chlorantraniliprole	7.29 × 10^−4^	1.03 × 10^−3^	5.98 × 10^−4^	8.45 × 10^−4^	2.88 × 10^−4^	4.07 × 10^−4^	5.74 × 10^−4^	8.10 × 10^−4^	2.59 × 10^−4^	3.66 × 10^−4^	2.94 × 10^−4^	4.15 × 10^−4^
Iprovalicarb	1.69 × 10^−2^	2.39 × 10^−2^	2.64 × 10^−2^	3.73 × 10^−2^	1.01 × 10^−2^	1.43 × 10^−2^	1.04 × 10^−1^	1.47 × 10^−1^	7.17 × 10^−2^	1.01 × 10^−1^	9.54 × 10^−2^	1.35 × 10^−1^
Myclobutanil	1.14 × 10^−2^	1.60 × 10^−2^	1.26 × 10^−2^	1.78 × 10^−2^	1.09 × 10^−2^	1.55 × 10^−2^	1.09 × 10^−2^	1.54 × 10^−2^	1.15 × 10^−2^	1.62 × 10^−2^	1.15 × 10^−2^	1.62 × 10^−2^
Tebuconazole	1.23 × 10^−2^	1.74 × 10^−2^	7.87 × 10^−3^	1.11 × 10^−2^	-	-	6.75 × 10^−3^	9.53 × 10^−3^	-	-	-	-
Oxathiapiprolin	2.18 × 10^−3^	3.08 × 10^−3^	2.02 × 10^−3^	2.85 × 10^−3^	-	-	-	-	-	-	-	-
Hazard Index (HI)	4.93 × 10^−2^	6.96 × 10^−2^	5.60 × 10^−2^	7.91 × 10^−2^	2.92 × 10^−2^	4.12 × 10^−2^	1.28 × 10^−1^	1.80 × 10^−1^	8.81 × 10^−2^	1.24 × 10^−1^	1.11 × 10^−1^	1.57 × 10^−1^

**Table 5:** The characteristics of the pesticide residues.

Pesticide Residues	Family-Activity	Pest Control	Molecular Weight	Log *P*	MRL Wines (ng/g)	EU Pesticides Data	Hazard Class and Category Code(s)
Acetamiprid	Neonicotinoid insecticide	Leafhoppers and other small insect pests	222.67	0.80	500	ADI = 0.025 mg/kg bw/day ARfD = 0.025 mg/kg bw AOEL = 0.025 mg/kg bw/day, Reg. (EU) 2018/113	H302: Harmful if swallowed Acute Tox. 4—Acute toxicity H412: Harmful to aquatic life with long lasting effects Aquatic Chronic 3—Hazardous to the aquatic environment
Chlorantraniliprole	Diamide insecticides		483.1	2.76	1000	ADI = 1.56 mg/kg bw/day ARfD = Not applicable AOEL = 0.36 mg/kg bw/day, EFSA 2013	No classification
Iprovalicarb	Carabamate fungicide and valinamide fungicide	Downey mildew	320.4	3.2	2000	ADI = 0.015 mg/kg bw/day AOEL = 0.015 mg/kg bw/day, Reg. (EU) 2016/147	No classification
Myclobutanil	Conazole fungicide	Powdery mildew	288.77	2.94	100	ADI = 0.025 mg/kg bw/day ARfD = 0.31 mg/kg bw AOEL = 0.03 mg/kg bw/day, EFSA 10	H302: Harmful if swallowed Acute Tox. 4—Acute toxicity H361d: Suspected of damaging the unborn child. Repr. 2—Reproductive toxicity H319: Causes serious eye irritation Eye Irrit. 2—Eye irritation H411: Toxic to aquatic life with long lasting effects Aquatic Chronic 2—Hazardous to the aquatic environment
Tebuconazole	Conazole fungicide	Powdery mildew	307.82	3.7	1000	ADI = 0.03 mg/kg bw/day ARfD = 0.03 mg/kg bw, EFSA 08 AOEL = 0.03 mg/kg bw/day, Dir 08/125	H302: Harmful if swallowed H400: Very toxic to aquatic life Aquatic Acute 1—Hazardous to the aquatic environment H361d: Suspected of damaging the unborn child. Repr. 2—Reproductive toxicity H410: Very toxic to aquatic life with long lasting effects Aquatic Chronic 1—Hazardous to the aquatic environment
Oxathiapiprolin	Fungicide	Oomycete pathogens	539.5	3.67	700	ADI = 0.14 mg/kg bw/day AOEL = 0.04 mg/kg bw/day, Reg. (EU) 2017/239	No classification

ADI: acceptable daily intake (mg/kg bw/day); ARfD: acute reference dose (mg/kg bw); AOEL: acceptable operator exposure level (mg/kg bw/day).

**Table 6:** The chronic (long-term) exposure of EU populations to pesticide residues from Romanian wines, expressed as the acceptable daily intake (ADI, in percent) and exposure (Exp, in µg/kg bw per day).

Active Substance	Wine Type	EFSA PRIMo rev.3.1	Population Group											
			PT General	FR Adult	RO General	IE Adult	UK Adult	DK Adult	DE Women	DE General	UK Vegetarian	NL General	ES Adult	FI Adult
Acetamiprid	Red wine	ADI (%)	0.7%	0.6%	0.5%	0.3%	0.3%	0.3%	0.2%	0.2%	0.2%	0.2%	0.1%	0.1%
		Exp (µg/kg bw per day)	0.17	0.16	0.12	0.09	0.08	0.07	0.06	0.06	0.06	0.04	0.03	0.02
	Rosé wine	ADI (%)	0.8%	0.7%	0.5%	0.4%	0.3%	0.3%	0.3%	0.3%	0.3%	0.2%	0.1%	0.1%
		Exp (µg/kg bw per day)	0.19	0.18	0.13	0.10	0.08	0.07	0.07	0.06	0.06	0.05	0.03	0.02
	White wine 1	ADI (%)	0.9%	0.9%	0.6%	0.5%	0.4%	0.4%	0.3%	0.3%	0.3%	0.2%	0.2%	0.1%
		Exp (µg/kg bw per day)	0.23	0.22	0.16	0.12	0.10	0.09	0.08	0.08	0.08	0.06	0.04	0.03
	White wine 2	ADI (%)	0.6%	0.6%	0.4%	0.3%	0.3%	0.2%	0.2%	0.2%	0.2%	0.1%	0.1%	0.1%
		Exp (µg/kg bw per day)	0.15	0.14	0.10	0.08	0.07	0.06	0.05	0.05	0.05	0.04	0.03	0.02
	White wine 3	ADI (%)	0.6%	0.5%	0.4%	0.3%	0.2%	0.2%	0.2%	0.2%	0.2%	0.1%	0.1%	0.1%
		Exp (µg/kg bw per day)	0.14	0.13	0.09	0.07	0.06	0.05	0.05	0.05	0.05	0.03	0.02	0.02
	White wine 4	ADI (%)	0.5%	0.5%	0.3%	0.3%	0.2%	0.2%	0.2%	0.2%	0.2%	0.1%	0.1%	0.1%
		Exp (µg/kg bw per day)	0.13	0.12	0.08	0.06	0.05	0.05	0.04	0.04	0.04	0.03	0.02	0.02
Chlorantraniliprole	Red wine	ADI (%)	0.1%	0.1%	0.1%	0.0%	0.0%	0.0%	0.0%	0.0%	0.0%	0.0%	0.0%	0.0%
		Exp (µg/kg bw per day)	1.36	1.27	0.92	0.68	0.59	0.52	0.46	0.45	0.44	0.32	0.23	0.17
	Rosé wine	ADI (%)	0.1%	0.1%	0.0%	0.0%	0.0%	0.0%	0.0%	0.0%	0.0%	0.0%	0.0%	0.0%
		Exp (µg/kg bw per day)	1.11	1.04	0.75	0.56	0.49	0.43	0.37	0.37	0.36	0.26	0.19	0.14
	White wine 1	ADI (%)	0.0%	0.0%	0.0%	0.0%	0.0%	0.0%	0.0%	0.0%	0.0%	0.0%	0.0%	0.0%
		Exp (µg/kg bw per day)	0.54	0.50	0.36	0.27	0.23	0.21	0.18	0.18	0.18	0.13	0.09	0.07
	White wine 2	ADI (%)	0.1%	0.1%	0.0%	0.0%	0.0%	0.0%	0.0%	0.0%	0.0%	0.0%	0.0%	0.0%
		Exp (µg/kg bw per day)	1.07	1.00	0.72	0.54	0.47	0.41	0.36	0.36	0.35	0.25	0.18	0.13
	White wine 3	ADI (%)	0.0%	0.0%	0.0%	0.0%	0.0%	0.0%	0.0%	0.0%	0.0%	0.0%	0.0%	0.0%
		Exp (µg/kg bw per day)	0.48	0.45	0.33	0.24	0.21	0.19	0.16	0.16	0.16	0.11	0.08	0.06
	White wine 4	ADI (%)	0.0%	0.0%	0.0%	0.0%	0.0%	0.0%	0.0%	0.0%	0.0%	0.0%	0.0%	0.0%
		Exp (µg/kg bw per day)	0.55	0.51	0.37	0.28	0.24	0.21	0.18	0.18	0.18	0.13	0.09	0.07
Iprovalicarb	Red wine	ADI (%)	2.0%	1.9%	1.4%	1.0%	0.9%	0.8%	0.7%	0.7%	0.7%	0.5%	0.3%	0.2%
		Exp (µg/kg bw per day)	0.30	0.28	0.20	0.15	0.13	0.12	0.10	0.10	0.10	0.07	0.05	0.04
	Rosé wine	ADI (%)	3.2%	2.9%	2.1%	1.6%	1.4%	1.2%	1.1%	1.1%	1.0%	0.7%	0.5%	0.4%
		Exp (µg/kg bw per day)	0.47	0.44	0.32	0.24	0.21	0.18	0.16	0.16	0.15	0.11	0.08	0.06
	White wine 1	ADI (%)	1.2%	1.1%	0.8%	0.6%	0.5%	0.5%	0.4%	0.4%	0.4%	0.3%	0.2%	0.1%
		Exp (µg/kg bw per day)	0.18	0.17	0.12	0.09	0.08	0.07	0.06	0.06	0.06	0.04	0.03	0.02
	White wine 2	ADI (%)	12.5%	11.6%	8.4%	6.3%	5.4%	4.8%	4.2%	4.2%	4.1%	2.9%	2.1%	1.5%
		Exp (µg/kg bw per day)	1.87	1.74	1.26	0.94	0.81	0.72	0.63	0.62	0.61	0.44	0.31	0.23
	White wine 3	ADI (%)	8.6%	8.0%	5.8%	4.3%	3.7%	3.3%	2.9%	2.9%	2.8%	2.0%	1.4%	1.1%
		Exp (µg/kg bw per day)	1.28	1.20	0.87	0.65	0.56	0.49	0.43	0.43	0.42	0.30	0.22	0.16
	White wine 4	ADI (%)	11.4%	10.6%	7.7%	5.7%	5.0%	4.4%	3.8%	3.8%	3.7%	2.7%	1.9%	1.4%
		Exp (µg/kg bw per day)	1.71	1.59	1.15	0.86	0.74	0.66	0.57	0.57	0.56	0.40	0.29	0.21
Myclobutanil	Red wine	ADI (%)	1.4%	1.3%	0.9%	0.7%	0.6%	0.5%	0.5%	0.5%	0.4%	0.3%	0.2%	0.2%
		Exp (µg/kg bw per day)	0.34	0.32	0.23	0.17	0.15	0.13	0.11	0.11	0.11	0.08	0.06	0.04
	Rosé wine	ADI (%)	1.5%	1.4%	1.0%	0.8%	0.7%	0.6%	0.5%	0.5%	0.5%	0.4%	0.3%	0.2%
		Exp (µg/kg bw per day)	0.38	0.35	0.25	0.19	0.16	0.14	0.13	0.13	0.12	0.09	0.06	0.05
	White wine 1	ADI (%)	1.3%	1.2%	0.9%	0.7%	0.6%	0.5%	0.4%	0.4%	0.4%	0.3%	0.2%	0.2%
		Exp (µg/kg bw per day)	0.33	0.30	0.22	0.16	0.14	0.13	0.11	0.11	0.11	0.08	0.05	0.04
	White wine 2	ADI (%)	1.3%	1.2%	0.9%	0.7%	0.6%	0.5%	0.4%	0.4%	0.4%	0.3%	0.2%	0.2%
		Exp (µg/kg bw per day)	0.33	0.30	0.22	0.16	0.14	0.12	0.11	0.11	0.11	0.08	0.05	0.04
	White wine 3	ADI (%)	1.4%	1.3%	0.9%	0.7%	0.6%	0.5%	0.5%	0.5%	0.4%	0.3%	0.2%	0.2%
		Exp (µg/kg bw per day)	0.34	0.32	0.23	0.17	0.15	0.13	0.12	0.11	0.11	0.08	0.06	0.04
	White wine 4	ADI (%)	1.4%	1.3%	0.9%	0.7%	0.6%	0.5%	0.5%	0.5%	0.4%	0.3%	0.2%	0.2%
		Exp (µg/kg bw per day)	0.34	0.32	0.23	0.17	0.15	0.13	0.12	0.11	0.11	0.08	0.06	0.04
Tebuconazole	Red wine	ADI (%)	1.5%	1.4%	1.0%	0.7%	0.6%	0.6%	0.5%	0.5%	0.5%	0.3%	0.2%	0.2%
		Exp (µg/kg bw per day)	0.44	0.41	0.30	0.22	0.19	0.17	0.15	0.15	0.14	0.10	0.07	0.05
	Rosé wine	ADI (%)	0.9%	0.9%	0.6%	0.5%	0.4%	0.4%	0.3%	0.3%	0.3%	0.2%	0.2%	0.1%
		Exp (µg/kg bw per day)	0.28	0.26	0.19	0.14	0.12	0.11	0.09	0.09	0.09	0.07	0.05	0.03
	White wine 1	ADI (%)	-	-	-	-	-	-	-	-	-	-	-	-
		Exp (µg/kg bw per day)	-	-	-	-	-	-	-	-	-	-	-	-
	White wine 2	ADI (%)	0.8%	0.8%	0.5%	0.4%	0.4%	0.3%	0.3%	0.3%	0.3%	0.2%	0.1%	0.1%
		Exp (µg/kg bw per day)	0.24	0.23	0.16	0.12	0.11	0.09	0.08	0.08	0.08	0.06	0.04	0.03
	White wine 3	ADI (%)	-	-	-	-	-	-	-	-	-	-	-	-
		Exp (µg/kg bw per day)	-	-	-	-	-	-	-	-	-	-	-	-
	White wine 4	ADI (%)	-	-	-	-	-	-	-	-	-	-	-	-
		Exp (µg/kg bw per day)	-	-	-	-	-	-	-	-	-	-	-	-
Oxathiapiprolin	Red wine	ADI (%)	0.3%	0.2%	0.2%	0.1%	0.1%	0.1%	0.1%	0.1%	0.1%	0.1%	0.0%	0.0%
		Exp (µg/kg bw per day)	0.36	0.34	0.25	0.18	0.16	0.14	0.12	0.12	0.12	0.09	0.06	0.05
	Rosé wine	ADI (%)	0.2%	0.2%	0.2%	0.1%	0.1%	0.1%	0.1%	0.1%	0.1%	0.1%	0.0%	0.0%
		Exp (µg/kg bw per day)	0.34	0.31	0.23	0.17	0.15	0.13	0.11	0.11	0.11	0.08	0.06	0.04
	White wine 1	ADI (%)	-	-	-	-	-	-	-	-	-	-	-	-
		Exp (µg/kg bw per day)	-	-	-	-	-	-	-	-	-	-	-	-
	White wine 2	ADI (%)	-	-	-	-	-	-	-	-	-	-	-	-
		Exp (µg/kg bw per day)	-	-	-	-	-	-	-	-	-	-	-	-
	White wine 3	ADI (%)	-	-	-	-	-	-	-	-	-	-	-	-
		Exp (µg/kg bw per day)	-	-	-	-	-	-	-	-	-	-	-	-
	White wine 4	ADI (%)	-	-	-	-	-	-	-	-	-	-	-	-
		Exp (µg/kg bw per day)	-	-	-	-	-	-	-	-	-	-	-	-

PT general—Portugal: general population; FR adult—France: adults ≥15 years; RO general—Romania: general population; IE adult—Ireland: adult, 18–64 years; UK Adult—United Kingdom of Great Britain: adult 19–64 years; DK adult—Denmark: adult 15–74 years; DE women—Germany: Women of child-bearing age, 14–50 years; DE general—Germany: general population; UK vegetarian—United Kingdom of Great Britain; NL general—Netherlands: general; ES adult—Spain: adult ≥17 years; FI adult—Finland: adult.

## Data Availability

The data presented in this study are available on request from the first author.

## References

[1] (2022). Organisation Internationale de la Vigne et du Vin. https://www.oiv.int/js/lib/pdfjs/web/viewer.html?file=/public/medias/8767/acitvityreporteng.pdf.

[2] Dumitriu D., Peinado R.A., Peinado J., de Lerma N.L. (2015). Grape pomace extract improves the in vitro and in vivo antioxidant properties of wines from sun light dried Pedro Ximénez grapes. J. Funct. Foods.

[3] Fermaud M., Smits N., Merot A., Roudet J., Thiery D., Wery J., Delbac L. (2016). New multipest damage indicator to assess protection strategies in grapevine cropping systems: An indicator of multipest damage in grapevine. Aust. J. Grape Wine Res..

[4] Simonovici M. (2019). Enquete Pratiques phytosanitaires en viticulture en 2016. Nombre de traitements et indicateurs de fréquence de traitement. https://www.agreste.agriculture.gouv.fr/agreste-web/download/publication/publie/Dos1902/Dossier2019-2.pdf.

[5] Jermini M., Blaise P., Gessler C. (2010). Quantitative effect of leaf damage caused by downy mildew (*Plasmopara viticola*) on growth and yield quality of grapevine “Merlot” (*Vitis vinifera*). J. Grapevine Res..

[6] Pons A., Mouakka N., Deliere L., Crachereau J.C., Davidou L., Sauris P., Guilbault P., Darriet P. (2018). Impact of *Plasmopara viticola* infection of Merlot and Cabernet Sauvignon grapes on wine composition and flavor. Food Chem..

[7] Merot A., Smits N. (2020). Does conversion to organic farming impact vineyards yield? A diachronic study in southeastern France. Agronomy.

[8] Pizzutti I.R., Scholten J., Righi L.W., Cardoso C.D., Rohers G.N., da Silva R.C. (2014). Development, optimization and validation of a multimethod for the determination of 36 mycotoxins in wines by liquid chromatography-tandem mass spectrometry. Talanta.

[9] Petit A.-N., Fontaine F., Clément C., Vaillant-Gaveau N. (2008). Photosynthesis Limitations of Grapevine after Treatment with the Fungicide Fludioxonil. J. Agric. Food Chem..

[10] Tsakirakis A.N., Kasiotis K.M., Charistou A.N., Arapaki N., Tsatsakis A., Tsakalof A., Machera K. (2014). Dermal & inhalation exposure of operators during fungicide application in vineyards. Evaluation of coverall performance. Sci. Total Environ..

[11] Raherison C., Baldi I., Pouquet M., Berteaud E., Moesch C., Bouvier G., Canal-Raffin M. (2018). Airborne Pesticide Exposure in Vineyard Rural Areas and Respiratory Health in Children: A pilot study. Environ. Res..

[12] Thierry D., Yengue J.-L. (2018). Vigne et ville. Le paradoxe de l′urbanisme. Rev. Des. Oenogues.

[13] Dumitriu (Gabur) G.-D., Teodosiu C., Cotea V.V., Morata A., Loira I., González C. (2021). Management of Pesticides from Vineyard to Wines: Focus on Wine Safety and Pesticides Removal by Emerging Technologies. Grapes and Wine.

[14] (2022). EC, European Commission-Farm to Fork Strategy. https://ec.europa.eu/food/plants/pesticides/sustainable-use-pesticides/farm-fork-targets-progress_en.

[15] Brancato A., Brocca D., Ferreira L., Greco L., Jarrah S., Leuschner R., Medina P., Miron I., Nougadere A., European Food Safety Authority (EFSA) (2018). Use of EFSA Pesticide Residue Intake Model (EFSA PRIMo revision 3). EFSA J..

[16] Romero-González R., Garrido Frenich A., Martínez Vidal J.L., Prestes O.D., Grio S.L. (2011). Simultaneous determination of pesticides, biopesticides and mycotoxins in organic products applying a quick, easy, cheap, effective, rugged and safe extraction procedure and ultra-high performance liquid chromatography–tandem mass spectrometry. J. Chromatogr. A.

[17] Cabras P., Angioni A. (2000). Pesticide residues in grapes, wine, and their processing products. J. Agric. Food Chem..

[18] Słowik-Borowiec M., Szpyrka E. (2018). Multiresidue Analysis of Pesticides in Wine and Grape Using Gas Chromatography with Microelectron Capture and Nitrogen–Phosphorus Detection. Food Anal. Methods.

[19] Anastasiades M., Lehotay S.J., Stajnbaher D., Schenck F.J. (2003). Fast and easy multiresidue method employing acetonitrile extrac- tion/partitioning and dispersive solid-phase extraction for the determination of pesticide residues in produce. J. AOAC Int..

[20] Dasgupta S., Banerjee K., Dhumal K.N., Adsule P.G. (2011). Optimization of Detection Conditions and Single-Laboratory Validation of a Multiresidue Method for the Determination of 135 Pesticides and 25 Organic Pollutants in Grapes and Wine by Gas Chromatography Time-of-Flight Mass Spectrometry. J. AOAC Int..

[21] Walorczyk S., Drozdzynski D., Gnusowski B. (2011). Multiresidue determination of 160 pesticides in wines employing mixed-mode dispersive-solid phase extraction and gas chromatography–tandem mass spectrometry. Talanta.

[22] Semla M., Schwarcz P., Mezey J., Binkowski Ł.J., Błaszczyk M., Formicki G., Gren A., Stawarz R., Massanyi P. (2018). Biogenic and risk elements in wines from the Slovak market with the estimation of consumer exposure. Biol. Trace Elem. Res..

[23] Dumitriu (Gabur) G.D., Teodosiu C., Morosanu I., Plavan O., Gabur I., Cotea V.V. (2021). Heavy metals assessment in the major stages of winemaking: Chemometric analysis and impacts on human health and environment. J. Food Compost. Anal..

[24] OIV (2019). Comparison of International Alcohol Drinking Guidelines. https://www.oiv.int/public/medias/7169/oiv-report-alcohol-drinking-guidelines-collective-expertise.pdf.

[25] Health Promotion and Disease Prevention Knowledge Gateway. https://knowledge4policy.ec.europa.eu/health-promotion-knowledge-gateway/national-low-risk-drinking-recommendations-drinking-guidelines_en.

[26] Gad Alla S.A., Loutfy N.M., Shendy A.H., Ahmed M.T. (2015). Hazard index, a tool for a long–term risk assessment of pesticide residues in some commodities, a pilot study. Regul. Toxicol. Pharmacol..

[27] Sharafi K., Yunesian M., Nodehi R.N., Mahvi A.H., Pirsaheb M. (2019). A systematic literature review for some toxic metals in widely consumed rice types (domestic and imported) in Iran: Human health risk assessment, uncertainty and sensitivity analysis. Ecotoxicol. Environ. Saf..

[28] European Commission (2019). Analytical Quality Control and Method Validation for Pesticide Residues Analysis in Food and Feed. https://ec.europa.eu/food/system/files/2020-01/pesticides_mrl_guidelines_wrkdoc_2019-12682.pdf.

[29] Drogue S., DeMaria F. (2012). Pesticide residues and trade, the apple of discord. Food Policy.

[30] Schusterova D., Hajslova J., Kocourek V., Pulkrabova J. (2021). Pesticide Residues and Their Metabolites in Grapes and Wines from Conventional and Organic Farming System. Foods.

[31] Cordova D., Benner E., Sacher M., Rauh J., Sopa J., Lahm G., Selby T., Stevenson T., Flexner L., Gutteridge S. (2006). Anthranilic diamides: A new class of insecticides with a novel mode of action, ryanodine receptor activation. Pest. Biochem. Physiol..

[32] Malhat F.M. (2012). Determination of Chlorantraniliprole Residues in Grape by High-Performance Liquid Chromatography. Food Anal. Methods.

[33] Caboni P., Sarais G., Angioni A., Vargiu S., Pagnozzi D., Cabras P., Casida J. (2008). Liquid chromatography–tandem mass spectrometric ion- switching determination of chlorantraniliprole and flubendiamide in fruits and vegetables. J. Agric. Food Chem..

[34] Angioni A., Dedola F., Garau A., Sarais G., Cabras P., Caboni P. (2011). Chlorpyrifos residues levels in fruits and vegetables after field treatment. J. Environ. Sci. Health B.

[35] Gonzalez-Rodrıguez R.M., Cancho-Grande B., Simal-Gandara J. (2009). Efficacy of new commercial formulations to control downy mildew and dissipation of their active fungicides in wine after good agricultural practices. J. Sci. Food Agric..

[36] Athanasopoulos P.E., Pappas C.J., Kyriakidis N.V. (2003). Decomposition of myclobutanil and triadimefon in grapes on the vines and during refrigerated storage. Food Chem..

[37] Carpinteiro I., Ramil M., Rodríguez I., Cela R. (2010). Determination of fungicides in wine by mixed-mode solid phase extraction and liquid chromatography coupled to tandem mass spectrometry. J. Chromatogr. A.

[38] Pelajic M., Pecek G., Pavlovic D.M., Cepo D.V. (2016). Novel multiresidue method for determination of pesticides in red wine using gas chromatography-mass spectrometry and solid phase extraction. Food Chem..

[39] Strickland T.C., Potter T.L., Joo H. (2004). Tebuconazole dissipation and metabolism in Tifton loamy sand during laboratory incubation. Pest Manag. Sci..

[40] Pasteris R.J., Hanagan M.A., Shapiro R.J.I. (2008). Fungicidal Azocyclic Amides.

[41] Pasteris R.J., Hanagan M.A., Bisaha J.J., Finkelstein B.L., Hoffman L.E., Gregory V., Andreassi J.L., Sweigard J.A., Klyashchitsky B.A., Henry Y.T. (2016). Discovery of oxathiapiprolin, a new oomycete fungicide that targets an oxysterol binding protein. Bioorgan. Med. Chem..

[42] Pingzhong Y., Jia C., Zhao E., Chen L., He H., Jing J., He M. (2017). Determination of oxathiapiprolin concentration and dissipation in grapes and soil by ultrahigh-performance liquid chromatography-tandem mass spectrometry. J. Sci. Food Agric..

[43] Wu X., Xu J., Dong F., Liu X., Li Y., Zheng Y. (2014). Simultaneous determination of oxathiapiprolin and two metabolites in fruits, vegetables and cereal using a modified quick, easy, cheap, effective, rugged, and safe method and liquid chromatography coupled to tandem mass spectrometry. J. Chromatogr. A.

[44] Sattler C., Kächele H., Verch G. (2007). Assessing the intensity of pesticide use in agriculture. Agr. Ecosyst. Environ..

[45] Golge O., Kabak B. (2018). Pesticide residues in table grapes and exposure assessment. J. Agric. Food Chem..

[46] WHO (1989). Guidelines for Predicting Dietary Intake of Pesticide Residues.

